# Superparamagnetic iron oxide nanoparticle attachment on array of micro test tubes and microbeakers formed on p-type silicon substrate for biosensor applications

**DOI:** 10.1186/1556-276X-6-540

**Published:** 2011-10-04

**Authors:** Sarmishtha Ghoshal, Abul AM Ansar, Sufi O Raja, Arpita Jana, Nil R Bandyopadhyay, Anjan K Dasgupta, Mallar Ray

**Affiliations:** 1 School of Materials Science and Engineering, Bengal Engineering and Science University, Shibpur, Howrah 711103, West Bengal, India; 2 Department of Biochemistry, Calcutta University, 35 Ballygunge Circular Road, Kolkata 700019, West Bengal, India

**Keywords:** porous silicon, SPION, biosensor

## Abstract

A uniformly distributed array of micro test tubes and microbeakers is formed on a p-type silicon substrate with tunable cross-section and distance of separation by anodic etching of the silicon wafer in N, N-dimethylformamide and hydrofluoric acid, which essentially leads to the formation of macroporous silicon templates. A reasonable control over the dimensions of the structures could be achieved by tailoring the formation parameters, primarily the wafer resistivity. For a micro test tube, the cross-section (i.e., the pore size) as well as the distance of separation between two adjacent test tubes (i.e., inter-pore distance) is typically approximately 1 μm, whereas, for a microbeaker the pore size exceeds 1.5 μm and the inter-pore distance could be less than 100 nm. We successfully synthesized superparamagnetic iron oxide nanoparticles (SPIONs), with average particle size approximately 20 nm and attached them on the porous silicon chip surface as well as on the pore walls. Such SPION-coated arrays of micro test tubes and microbeakers are potential candidates for biosensors because of the biocompatibility of both silicon and SPIONs. As acquisition of data via microarray is an essential attribute of high throughput bio-sensing, the proposed nanostructured array may be a promising step in this direction.

## Introduction

The promotion of silicon (Si) from being the key substrate material for microelectronic devices to a potential light emitter emerged as a consequence of the possibility to reduce its dimension by different techniques [1-3]. Extensive research in this field was triggered after the discovery of light emission from electrochemically etched porous Si [1]. Research on porous Si has so far been primarily focused on microporous Si which have average pore diameter ≤2 nm [4], exhibit room temperature photoluminescence (PL) and consequently hold immense promise for potential light sources in opto-electronic devices. However, macroporous Si with typical pore diameters > 50 nm [4], do not exhibit PL but has found niche applications in the field of photonics [5], sensor technology and biomedicine [6,7]. Macroporous Si can potentially be used as a sensitive transducer material for detection of various biological and non-biological samples as its conductivity, capacitance, and/or refractive index changes upon adsorption of molecules on its surface [8,9]. Porous Si can also be permeated by different molecules leading to specific properties depending on the deposited substance and their morphology [10,11]. Because of its non-invasive and non-radioactive nature, porous Si promises versatile applications in medical diagnostics, pathogen detection, gene identification, and DNA sequencing [11,12]. The non-toxic behavior of porous Si makes it particularly suitable for biosensor applications including drug delivery platform for *in vivo *applications [10,13]. Extensive reviews on the scope of porous Si in nanobiotechnology have been reported in the literature [6,11,14].

For biological applications, porous Si structures with ordered arrangement of pores having diameters approximately 1 μm are desirable for loading molecules and drugs within the pores. Uniform macropore formation and its dependence on the formation parameters have been well reported [15,16]. Fewer Fabry-Perot fringes were observed for porous Si sensors fabricated at higher current densities because of greater porosity leading to matte surface [17]. Thus, engineering a uniform structure of macropores (approximately 1 μm in diameter), each of which appears as a micro test tube is very desirable for building porous Si-based biochips or biosensors. In addition, porous Si is known to be a suitable material for implementing an efficient and reliable surface-enhanced Raman scattering (SERS) substrate that can be used to detect the presence of chemical and biological molecules [18,19]. However, to make an SERS substrate, complete filling of the pores is undesirable as the exposed surface area is reduced and thus the target molecule may simply attach on the top surface. Nano-sized Si pillars (< 100 nm in width) with comparatively larger pores (> 1.5 μm in diameter), appear as microbeakers on porous Si, which provide a very convenient platform for SERS substrate. These microbeakers can be coated completely without filling the pores for various bio-sensing applications.

In first part of this work, we report fabrication of arrays of micro test tubes and microbeakers formed on p-type Si substrate with varying pore and particle sizes. For the micro test tubes, the pore size as well as the inter-pore distance is typically 1 μm (approximately), whereas, for a microbeaker the pore size exceeds 1.5 μm and the inter-pore distance could be less than 100 nm. Even with very thin Si walls, the microbeakers were found to be quite stable under ambient conditions. In the next part of this work, we successfully synthesized and attached superparamagnetic iron oxide nanoparticles (SPIONs) on the porous Si surface as well as on the pore walls using a simple and cost-effective technique. SPIONs have demonstrated their utility as non-invasive molecular probes to monitor biological processes, particularly by enhancing magnetic resonance (MR) contrast in MR imaging which allows monitoring of anatomical changes as well as physiological and molecular changes [20,21]. Therefore, such robust micro test tubes and microbeakers formed on Si substrates with SPION attachment promises to have immense applications in biomedicine and biomedical sensing due to biocompatible nature of both the materials [22,23].

### Experimental

Macroporous Si were formed on (100) orientation, p-type Si wafers in a specially designed teflon bath by anodic etching in hydrofluoric acid (HF) and N, N-dimethylformamide (DMF) solution. To obtain porous Si with different morphology, wafers of varying resistivity (*ρ*) ranging from 0.01 to 100 Ω-cm were used. The concentration ratios of HF/DMF, formation current density (*J*), etching time (*t*) were also varied to obtain porous layers having different porosity. SPIONs were synthesized by chemical co-precipitation of ferrous and ferric ion. Briefly, ferric and ferrous chlorides were dissolved in 2 M HCl in 2:1 (*w/w*) ratio and bare iron oxide was obtained by addition of 1.5 M NaOH. All steps were performed under nitrogen environment. The formed black precipitate was washed several times by de-ionized (DI) water through magnetic decantation to remove excess ions. Then the precipitate was re-dispersed in citrate buffer of pH 4 and finally pH was adjusted to 7 to form aqueous stable colloidal SPION solution. The as-synthesized SPIONs were loaded onto the desired porous Si chips by placing the porous template in a dense aqueous solution of SPIONs under magnetic incubation for 24 h. An external magnetic field of 70 Gauss was applied so as to drive the SPIONs inside the pores. This was repeated twice, first without disturbing the system and secondly, by spraying DI water on the chip at certain intervals during magnetic incubation so that the particles can penetrate inside the pores without adhering on the surface only, due to drying up of the aqueous SPION solution.

Macroporous Si samples (with and without SPION attachment) were investigated with the scanning electron microscope (SEM). The SEM used in the present study is a Hitachi S-3400N. The variable pressure mode of the instrument allowed investigation of the semiconducting samples in their natural state without the need of conventional sample preparation and coating. The microscope was operated at 20 to 30 kV and 10 to 5 mm working distance under variable pressure. Elemental analyses (qualitative) were done from the energy dispersive X-ray (EDX) spectra. Dynamic light scattering (DLS) and laser Doppler velocimetry (LDV), for determining the hydrodynamic size and the zeta potential respectively of the as-synthesized SPIONs in solution, were performed on a Malvern Instruments Zetasizer (5 mW HeNe laser, *λ *= 632 nm). The operating procedure was programmed such that there were averages of 25 runs, each run being averaged for 15 s, with an equilibration time of 3 min at 25°C. The magnetic properties of the SPIONs were investigated using a superconducting quantum interference device magnetometer (Model: MPMS-Quantum Design7).

## Results and discussions

### Formation of micro test tubes and microbeakers

**Figure 1:**
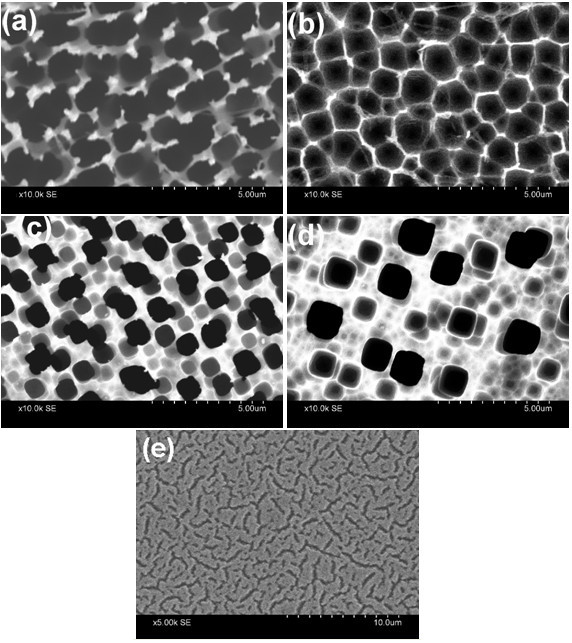
**Top-view SEM images of macroporous Si formed on p-type substrate with different formation parameters**. **(a) **random, wide, and connected porous structure formed on 0.1 to 0.5-Ω-cm wafer with *J *= 2 mA/cm^2^, *t *= 30 min and HF/DMF ratio = 1:11; **(b) **hexagonal, honey-comb type pore structure with narrow pore walls formed on 2 to 5-Ω-cm resistivity wafer using *J *= 3 mA/cm^2^, *t *= 60 min and HF/DMF ratio = 1:10; **(c) **more-or-less regular and circular macropores on 10-Ω-cm wafer formed with *J *= 5 mA/cm^2^, *t *= 60 min and HF/DMF ratio = 1:9; **(d) **widely separated pores formed with the same formation parameters as in (c) but on a 100-Ω-cm wafer; and **(e) **shows the formation of cracks without any resolvable porous structure for 0.01 to 0.05-Ω-cm wafer.

The variation of pore diameter and depth of pores in macroporous Si formed on p-type substrate with varying current density, etching time, and HF/DMF ratio is well studied [5,15,16]. We carried out a series of experiments by varying all the formation parameters including wafer resistivity over five orders of magnitude (0.01 to 0.05 Ω-cm, 0.1 to 0.5 Ω-cm, 2 to 5 Ω-cm, 10, and 100 Ω-cm). We found that macropore formation can be obtained for all the wafers (except for the most conductive one), by suitably tuning the current density and HF/DMF ratio as shown in Figure 1a, b, c, d. When the substrate resistivity is reduced to 0.01 to 0.05-Ω-cm macropore formation could not be observed for any attempted combination of current density and HF/DMF ratio. In most cases, homogeneous layers with resolvable cracks are observed as shown in Figure 1e. The findings suggest that there is a critical value of substrate resistivity (approximately 0.1 to 0.2 Ω-cm) below which no macropore is obtained for our samples and these observations are in agreement with those reported by Harraz et al. [16].

Several models regarding the mechanism of formation of macropores on p-type Si has so far been reported. The depletion and field effects model proposed by Lehmann and Rönnebeck [24], the chemical passivation model [25], the current burst model [26], etc. have been widely used, but a real consensus in this matter is still awaited. However, before commenting on the probable mechanism governing pore formation, we first note the major observations generated in this study with respect to the effect of wafer resistivity on pore morphology, which is partly reflected in the images shown in Figure 1: (1) the thickness of the macropore walls are greatly reduced with decrease in resistivity of the starting substrate; (2) for given current density and HF/DMF ratio, inter-pore spacing increases but the pore density decreases with increase in resistivity of the substrate; (3) the pore diameter also decreases with decreasing resistivity (though on comparing Figure 1a with either c or d this might seem contradictory, one has to note that the voids seen in Figure 1a are due to more than one interconnected pores); (4) there is probably some critical threshold resistivity (approximately 0.1 to 0.2 Ω-cm in our case) below which no macropore can be obtained; and (5) the geometry of the cross-section of the pore (roughly circular or hexagonal or rectangular) can be tailored by choosing different resistivity wafers. In addition, we also observed, in agreement with previous reports [5,15,16] that for a wafer of given resistivity, the pore diameter increases almost linearly with formation current density, whereas etching time primarily governs the pore-depth. The effect of HF concentration and HF/DMF ratio is relatively complex and is discussed elsewhere [16]. The presence of DMF in the electrolyte plays an important role in the formation process as it is a very good solvent for positive charge carriers [27]. The high concentration of DMF increases hole current at the pore walls causing widening of the pores. Therefore, for the low resistivity (*ρ *= 0.1 to 0.5 and 2 to 5 Ω-cm) samples, porous structure could be obtained only when both the current density and HF/DMF ratio were maintained at lower values.

Since the purpose of this work is to synthesize array of micro test tubes and microbeakers of Si for biological applications, and not on investigating the pore formation mechanism in p-Si, we refrain from making any assertive comments on this controversial issue. However, from the above observations, it seems likely that charge-transfer mechanisms similar to that of a Schottky diode in case of anodic etching of p-Si, in which case the holes migrate through the wafer towards the electrolyte/Si interface where the space charge region is formed, as suggested by the model of Lehmann and Rönnebeck [24], is in all possibility the dominant mechanism. The more-or-less square-root dependence of pore wall thickness on resistivity provides initial support to this model, whereas the variation of geometry of cross-section of the pore is suggestive of non-linear dissolution kinetics. A detailed analysis of the mechanism would no doubt depend on the systematic investigation of the role of each formation parameter and their interdependence, which warrants a separate investigation. Therefore, we focus only on the samples shown in Figure 1c, d for synthesis of microbeakers and micro test tubes.

**Figure 2:**
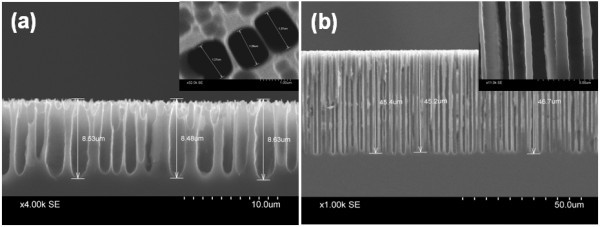
**Cross-sectional view of macroporous Si**. Showing **(a) **an array of microbeakers with depth approximately 8.5 μm and cross-sectional diameter approximately 1.5 μm formed on a 100 Ω-cm wafer, with *J *= 5 mA/cm^2^, *t *= 90 min and HF/DMF ratio = 1:9 and **(b) **an array of micro test tubes having length approximately 45 μm grown on a 10 Ω-cm wafer with the same parameters as mentioned in (a). The inset shown in (a) is the top-view of the sample showing regular pores thereby revealing that the apparent irregularity of the top surface of the cross-sectional view is introduced during cutting the sample in order to obtain the cross-sectional image. The inset shown in (b) reveals regular nature of the pores running almost parallel to each other with pore size as well as the inter-pore distance typically approximately1 μm.

Based on the observations reported above we synthesized array of micro test tubes and microbeakers on p-Si substrate by suitably choosing the formation parameters. The cross-sectional SEM images shown in Figure 2a, b clearly reveal the formation of such micro test tubes and microbeakers.

From the SEM image shown in Figure 2a, it is clear that microbeakers are formed on p-Si with distinct large pores having diameter around 1.5 μm along with very narrow inter-pore Si walls (approximately 100 nm). Whereas, Figure 2b reveals that a regular array of micro test tubes with length exceeding 45 μm and inter-pore distances around 1 μm is also obtainable on p-Si substrate. From the discussion presented before, it is obvious that the length of the pores in both cases can be controlled primarily by tailoring the etching time while the pore diameter, pore density, and consequently the inter-pore distances are easily controlled by varying the formation current density and HF/DMF ratio. This allows us to synthesize arrays of microbeakers and micro test tubes on p-Si substrate with desired lengths and cross-sections by suitably tuning the formation parameters.

### Superparamagnetic iron oxide nanoparticles

**Figure 3:**
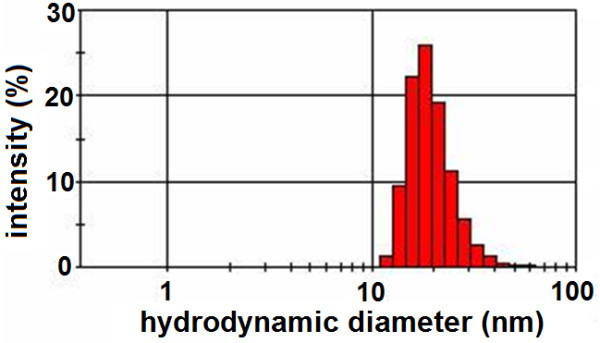
**Size distribution of the as-synthesized SPIONs obtained from DLS measurements shows maxima at 20 nm**.

The average hydrodynamic size of the as-synthesized SPIONs was measured by DLS study. DLS analyzes the velocity distribution of particle movement by measuring dynamic fluctuations of light-scattering intensity caused by the Brownian motion of the particle. This technique yields a hydrodynamic radius, or diameter, which is calculated using the Stokes-Einstein equation from the aforementioned measurements. The average particle size estimated in this manner is found to be approximately 20 nm as shown in Figure 3. The LDV-based zeta potential measurement of these SPIONs using a 5 mW He-Ne, 632-nm laser revealed that they have considerably high zeta potential value of -50 mV, which is an evidence of high colloidal stability [28].

**Figure 4:**
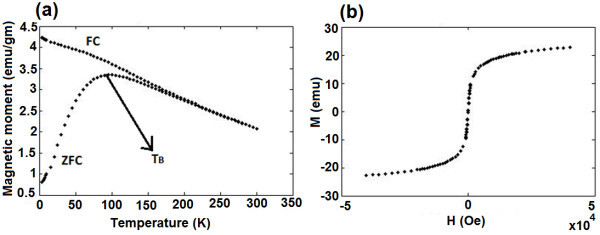
**FC/ZFC curves obtained at different temperatures and lack of hysteresis at room temperature**. **(a) **FC at 100 Oe and ZFC show a bifurcation and the maximum magnetic moment in ZFC provides an estimate of the blocking temperature (*T*_B_), which is approximately 100 K; and **(b) **M-H curve at 300 K shows no hysteresis.

The SPIONs were investigated in terms of field cooling (FC) and zero field cooling (ZFC) magnetization curves and hysteresis loops (M-H curves). The FC/ZFC curves obtained at different temperatures shown in Figure 4a clearly shows the presence of blocking temperature (*T*_B_) around 100 K. On the other hand, the lack of hysteresis at room temperature is evident from Figure 4b. The observation of superparamagnetic blocking and the absence of magnetic remanence directly demonstrate that the samples are superparamagnetic at room temperature [29].

### SPION attachment on macroporous silicon

**Figure 5:**
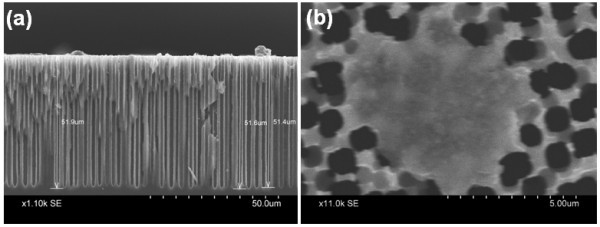
**SEM images of SPION attachment on array of micro test tubes**. **(a) **and **(b) **are the cross-sectional and top view respectively, showing substantial deposits of agglomerated particles.

In an attempt to render the array of micro test tubes and microbeakers as a potential biosensor, attempt was made to attach the as-synthesized SPIONs onto the porous template. The SEM images shown in Figure 5a, b clearly show the presence of SPIONs attached on the top surface of porous Si sample in the form of agglomerated clusters as well as inside the upper portion of the pores.

**Figure 6:**
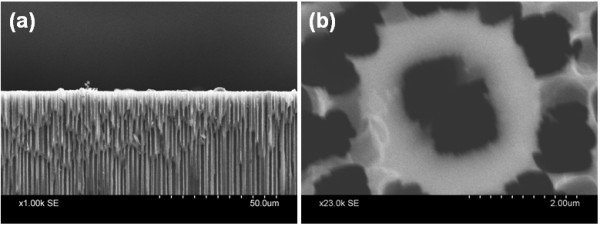
**SEM images of SPION attached micro test tubes following sprinkling of water during magnetic incubation**. **(a) **and **(b) **are the cross-sectional and top view, respectively.

A comparison of Figures 1c and 2b with Figure 5a explicitly reveals that magnetic incubation of the bare porous Si template has indeed resulted in SPION impregnation/attachment, primarily on the surface of the micro test tubes. From Figure 5a, b, it appears that the nanoparticles remain attached only on the upper portion of the pore walls with no trace at the bottom of the pore. We suspect that this happens as a result of drying up of the aqueous SPION solution during the process of magnetic incubation causing deposition of the particles mostly on the surface of the template. So, we repeated the process with frequent addition of water to prevent the solution from dehydrating. Figure 6a, b shows that the simple process of frequent sprinkling of DI water has helped in a comparatively better penetration of the SPIONs. Comparison of Figures 5b and 6b also show that keeping the solution hydrated has resulted in unblocking the pore though much of the SPIONs still reside on the surface. Furthermore, simple visual inspection of Figures 5a and 6a also suggests that water treatment has allowed the SPIONs to penetrate a greater depth through the pores and attach to the walls of Si.

**Figure 7:**
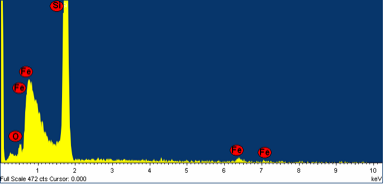
**SEM-EDX spectrum of the sample shown in Figure 6 indicating the presence of Fe**.

Finally, to cross-verify the presence of SPIONs in the porous Si samples, EDX spectra of the SPION-treated sample were obtained and one such spectrum is presented in Figure 7. The EDX spectrum shows clear peaks of Fe which establishes that the sample under investigation does have SPIONs. It may be noted here that similar experiments were performed with the microbeakers and it was relatively easier to get the SPIONs inside the pores because of the larger pore sizes and smaller inter-pore distances. However, the SPIONs tend to attach to the surface instead of penetrating into the pores when the aqueous solution dries up. The results are very similar to the ones presented in Figures 3 and 4 and hence not presented here. Attempts are now in progress to load the SPIONs in micro test tubes and microbeakers along with designed sequences of DNA at specific ensemble of the nanopores in an attempt to upgrade the system to a nano-designed array for specific biological applications.

## Conclusions

In summary, we have demonstrated successful fabrication of a uniformly distributed array of micro test tubes and microbeakers on p-type Si substrates with tunable dimensions. Iron oxide nanoparticles, with average particle size approximately 20 nm, synthesized using chemical co-precipitation and exhibiting superparamagnetic characteristics, were attached to the surface and to the walls of these micro test tubes and microbeakers without completely filling the pores. Such robust and cost-effective SPION attached micro test tubes and microbeakers formed on Si substrates have immense applications in biomedical sensing due to biocompatible nature of both the materials. By loading such SPIONs with designed sequences of DNA at specific ensemble of the nanopores may upgrade the system to a nano-designed array, the specific details of which is presently under progress.

## Competing interests

The authors declare that they have no competing interests.

## Authors' contributions

SG, AAMA, AJ, NRB, MR were all involved with the preparation of the micro test tubes and microbeakers on p-Si and analyses of the results. SEM imaging was performed by AJ and MR. SOR and ADG concentrated on the synthesis of SPIONs, magnetic characterization, and interpretation of results. The magnetic incubation and loading of SPIONS were carried out by SOR, AAMA, and SG. The idea of the present study was generated by SG, ADG, and MR. SG and MR collated all the results and drafted the paper. ADG also helped in drafting the final paper. All authors read and approved the final manuscript.
